# A Novel Classification System of Skinboosters to Support Clinical Decision Making

**DOI:** 10.1111/jocd.70945

**Published:** 2026-06-08

**Authors:** Gi‐Woong Hong, Isabella Rosellini, Hyunggyu Kang, Olena Sydorchuk, Kyu‐Ho Yi

**Affiliations:** ^1^ Samskin Plastic Surgery Clinic Seoul Korea; ^2^ Avery Beauty Clinic Malang Indonesia; ^3^ Medical Research Inc. Wonju Korea; ^4^ You and I Clinic Seoul Republic of Korea

## 1 Introduction

Skin boosters represent a revolutionary advancement in dermatological treatments, encompassing a diverse range of products designed to improve skin quality, hydration, and overall appearance through various mechanisms of action [1]. These innovative treatments have transformed the landscape of aesthetic medicine by moving beyond traditional approaches focused solely on volume augmentation to address the fundamental aspects of skin aging and deterioration [2]. Aging skin is largely characterized by a decline in key structural components—most notably hyaluronic acid (HA) and collagen—which leads to diminished skin elasticity and overall deterioration in skin quality [3, 4]. Consequently, many skinbooster formulations are designed to replenish these components of the extracellular matrix (ECM), targeting the restoration of HA and collagen as a central mechanism of action.

Although skinboosters encompass a wide variety of products, they can be systematically classified based on their primary effects and main source materials to provide clinical practitioners with a comprehensive framework for treatment selection and patient care. Based on the main source material, skinboosters can be categorized into five distinct groups: Autologous materials (derived from the patient's own blood or adipose tissue, such as platelet‐rich plasma and stem cell‐derived components), allogeneic materials (including human fibroblasts, allogeneic stem cells, exosomes, secretomes, cytokines, and collagen from human donors), xenogeneic materials (cross‐species grafts such as hyaluronic acid produced by microbial fermentation, polydeoxyribonucleotide extracted from salmon sperm, and chitosan (Arche, Doum Inc., Korea) derived from mushrooms), synthetic materials (man‐made polymers including collagen‐stimulating agents like Sculptra, Juvelook, Sihler P, and Facetem), and compound formulations (combination products such as mesotherapy cocktails that blend multiple active ingredients for synergistic therapeutic effects) [5, 6, 7].

Complementing this material‐based classification system, skinboosters can also be categorized according to their primary therapeutic effects into three fundamental types: Hydration‐based products that leverage hyaluronic acid's remarkable capacity to bind and retain water molecules while stimulating fibroblast activation and collagen synthesis, collagen‐based products that either stimulate endogenous collagen production through controlled inflammatory responses or provide exogenous collagen directly to enhance skin elasticity and firmness, and overall skin regeneration products that target multiple components of the skin's complex structure, including cells, extracellular matrix, and the dermal–epidermal junction, to achieve comprehensive skin rejuvenation and repair [8]. A thorough understanding of the primary components and the origins of raw materials in each product enables clinicians to infer the principal therapeutic effects and to anticipate potential safety concerns, while knowledge of how the main ingredient is sourced and processed is critical for proper clinical application, regulatory compliance, and addressing ethical and religious considerations that may vary across different patient populations and international markets.

## 2 Classification of Skinboosters

Although skinboosters encompass a wide variety of products, they can generally be classified based on their primary effects and main source materials.

**FIGURE 1:**
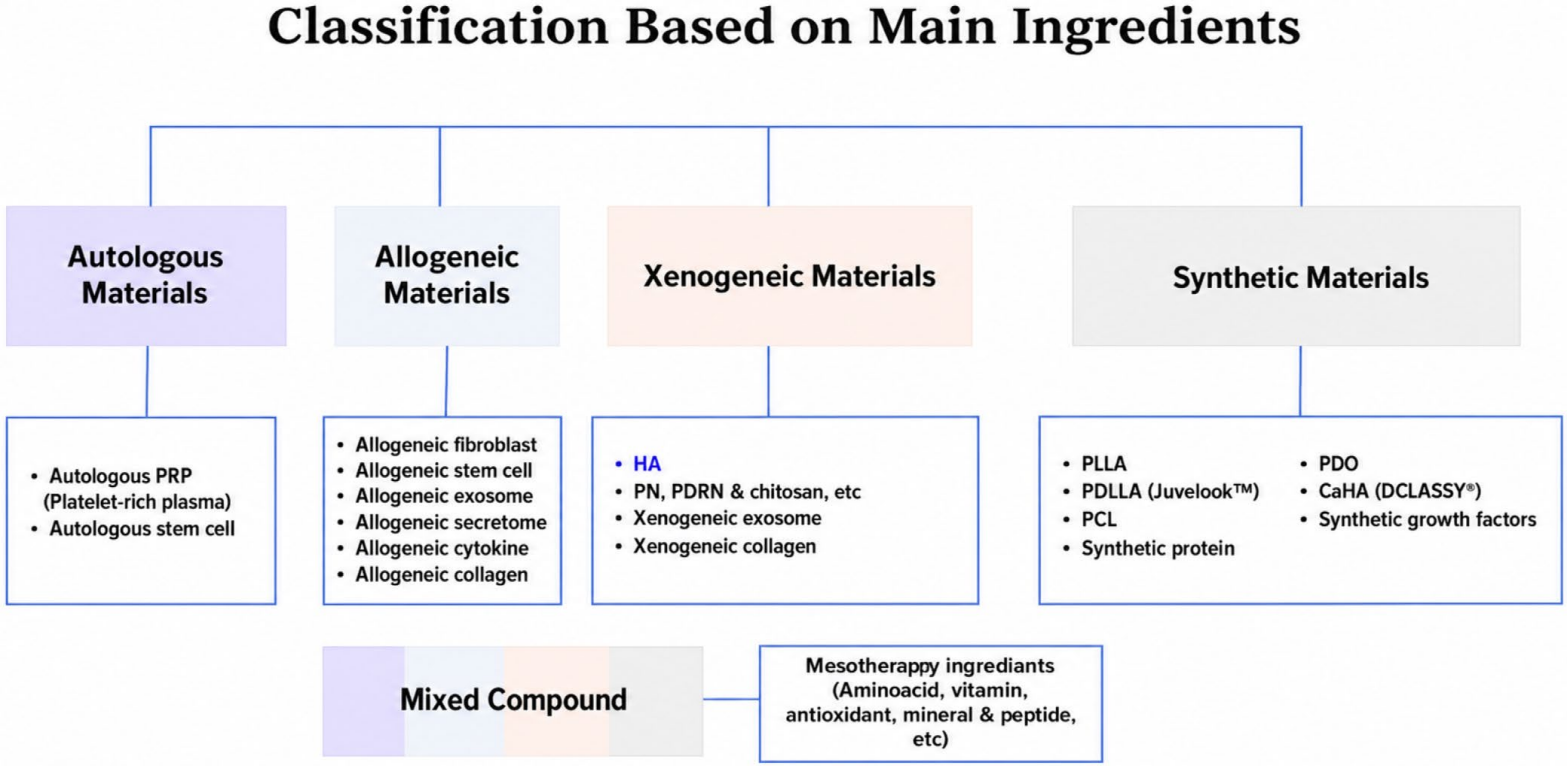
Classification of Skinboosters Based on Primary Source Material.

Based on the main source material, skinboosters can be categorized into five groups, as illustrated in Figure 1:

1. Autologous Materials: Autologous materials in dermatology refer to tissues, cells, or tissue‐engineered constructs derived from the same individual who will receive the treatment. This approach leverages the body's natural healing mechanisms while avoiding complications associated with foreign tissue transplantation, such as immune rejection, disease transmission, and ethical concerns related to donor tissue availability [4].

The fundamental principle underlying autologous therapy is histocompatibility—the biological compatibility between the donor tissue and the recipient site, which are essentially the same organism. This compatibility ensures optimal integration, survival, and functional restoration of the transplanted material [4].

These are the oldest types of skin boosters, derived from the patient's own blood or adipose tissue. Examples include *platelet‐rich plasma (PRP)* and *stem cell–derived components* obtained from autologous tissues.

2 Allogeneic Materials: Allogeneic materials represent a significant advancement in dermatological treatment, particularly in the management of severe skin injuries and disorders. These materials, derived from donors of the same species as the recipient, offer unique therapeutic advantages while presenting specific clinical considerations that must be carefully managed. These materials sometimes involve a cadaveric donor [3]. They include *human fibroblasts*, *allogeneic stem cells*, *allogeneic exosomes*, *secretomes*, *cytokines*, and *collagen*. Ethical and religious considerations may arise when using allogeneic materials.
3 Xenogeneic Materials: Xenogeneic extracellular matrix (ECM) materials are defined as biological grafts where the donor and recipient are from different species. In dermatological applications, this approach involves the transplantation of processed tissue materials from animal sources to human patients, creating a cross‐species therapeutic intervention that can provide structural and functional support for wound healing and tissue reconstruction. These cross‐species grafts have emerged as viable alternatives when human tissue sources are limited or unavailable, offering unique therapeutic possibilities while presenting distinct clinical and ethical considerations [3]. Representative examples include *hyaluronic acid (HA)* produced by microbial fermentation, *polydeoxyribonucleotide (PDRN)* and *polynucleotide (DOT‐PN, Rejuran, Korea)* extracted from salmon sperm, *chitosan* derived from mushroom (Arche, Doum, Korea), as well as *xenogeneic exosomes* and *collagen*.
4 Synthetic Materials: Synthetic materials in dermatology encompass a broad category of man‐made polymers specifically engineered for applications in skin substitutes and wound dressings. These materials are meticulously designed to address and overcome the inherent limitations often associated with natural polymers, such as their comparatively low mechanical strength and rapid degradation rates. By leveraging advanced polymer science, synthetic materials offer a superior combination of properties, making them highly suitable for the complex demands of skin wound treatment and regeneration [5, 6]. Examples include collagen‐stimulating agents such as *Sculptra (PLLA)*, *Juvelook (PDLLA)*, *Lafullen (PCL)*, *UltracoL (PDO)*, and *Facetem (CaHA)*.
5 Compound Formulations: Compounded formulations in dermatology represent a highly specialized and patient‐centric approach to medication, defined as the meticulous process of combining, mixing, or altering the ingredients of a drug by a licensed pharmacist (or under their direct supervision) or a licensed physician. These are combination products formulated with various materials—autologous, allogeneic, xenogeneic, or synthetic—designed to maximize therapeutic effect. This practice is fundamentally about creating a medication that is precisely tailored to the unique and individual needs of a patient, moving beyond the limitations of mass‐produced pharmaceutical products [7]. A representative example includes mesotherapy cocktails, which contain multiple active ingredients blended for synergistic benefit. Table 1 summarizes the proposed classification, representative ingredients/products, typical clinical indications, and key safety/ethical considerations.

**TABLE 1:** Summarizes the proposed classification, representative ingredients/products, typical clinical indications, and key safety/ethical considerations.

Primary source category	Representative ingredients/products (examples)	Dominant effect category	Typical clinical targets	Key safety/ethical considerations	Key supporting literature (examples)
Autologous	Platelet‐rich plasma (PRP); adipose‐derived cell preparations	Hydration/regeneration adjunct	Skin texture; fine lines; post‐procedure recovery	Procedure variability; standardization; infection control	Add appropriate PRP/ADSC clinical references (to be added)
Allogeneic	Human‐derived fibroblast/cell products; extracellular vesicles/exosomes (Sihler X); conditioned media/secretome	Overall skin regeneration	Photoaging; texture; multi‐parameter skin quality	Product standardization; regulatory status; donor screening	EV/CM systematic review (Jafarzadeh 2025); fibroblast grafting (Hirose 2024)
Xenogeneic	Fish‐derived PN/PDRN; Mushroom‐derived chitosan; animal‐derived collagen/ECM derivatives	Regeneration/repair	Skin quality support; adjunct in rejuvenation	Allergy risk; patient preference; ethical/religious issues	PN review (Lee [9]); PDRN review (Khan [10].)
Biotechnologically derived (if you prefer to mention within “compound” note)	HA produced by microbial fermentation (intradermal HA gels)	Hydration‐based	Hydration; skin smoothness; fine lines	Injection technique–dependent outcomes; ISRs; contraindications	VYC‐12 prospective study (Niforos 2019); VYC‐12 L biophysical study (Safa 2022)
Synthetic (biostimulatory fillers)	PLLA (Sculptra/others); CaHA (Radiesse); PCL (Lafullen); PDO (UltracoL); PDLLA (Juvelook/others)	Collagen‐based (biostimulation)	Laxity; dermal thinning; atrophic changes	Product‐specific AE profiles; nodules; technique sensitive	CaHA narrative review (Aguilera 2023); PLLA systematic review (Polymers 2024)
Compound formulations	Mesotherapy cocktails (multi‐ingredient blends)	Variable (often regeneration adjunct)	Dullness; mixed concerns; adjunct protocols	Regulatory status varies; component interactions	Add formulation‐specific references (to be added)
HA + humectant formulation	CPM‐HA20G + glycerol (e.g., Belotero Revive)	Hydration‐based	Hydration; barrier‐related skin quality	ISRs; technique; product availability by region	Hertz‐Kleptow 2019
Hybrid HA cooperative complexes	Stable hybrid cooperative complexes (e.g., Profhilo, Kyloe (Baren Inc., Korea))	Hydration/regeneration‐leaning	General skin quality; elasticity perception	Product positioning differs by region; post‐market safety	Salti 2025; Cheng 2025
Extracellular vesicles/exosomes	Exosome (Sihler X)‐based approaches (topical/intradermal, variable regulation)	Regeneration‐focused	Inflammation modulation; texture; rejuvenation claims	Standardization; contamination risk; regulatory constraints	Exosome review (Sreeraj 2024); EV/CM systematic review (Jafarzadeh 2025)
Polynucleotide family	Polynucleotide (DOT‐PN, Rejuran, Korea)	Regeneration‐focused	Texture; elasticity support; adjunct	Source disclosure; patient preference	PN review (Lee [1])
Polydeoxyribonucleotide	PDRN	Regeneration‐focused	Skin quality support; repair claims	Evidence heterogeneity; product differences	PDRN review (Khan [10])

Table 2 further summarizes the material‐origin classification of skinboosters, highlighting representative agents, key advantages, and major safety, regulatory, and ethical considerations for each source‐material category.

**TABLE 2:** Material‐origin classification + safety/regulatory/ethical.

Source‐material category	Representative agents	Key advantages	Safety/regulatory/ethical flags (examples)
Autologous	PRP, ADSC/SVF	Low immunogenicity, patient acceptance	Protocol variability; processing standards
Allogeneic	Human fibroblast‐derived products, allogeneic stem cell derivatives, exosomes/secretome, human collagen	Off‐the‐shelf, potent signaling	Donor/processing traceability; regulation differs by country; ethical/religious concerns
Xenogeneic	PN/PDRN (salmon), chitosan (mushroom), animal collagen	Availability, bioactivity	Allergy (e.g., mushroom/fish/shellfish), cultural/religious acceptance; theoretical pathogen concerns
Synthetic	PLLA/PDLLA/PCL/PDO/CaHA	Predictable manufacturing, durability	Irreversibility (vs. HA), adverse event management; technique‐dependent
Compound formulations	Mesotherapy cocktails, combinations	Synergistic targeting	Component‐level approval/labeling differs; “cocktail” variability

A thorough understanding of the primary components and the origins of raw materials in each product enables clinicians to infer the principal therapeutic effects and to anticipate potential safety concerns. For instance, in collagen‐based formulations, natural collagen contains telopeptides, which have been associated with immunogenic responses. In contrast, atelocollagen derived from fish undergoes enzymatic treatment to remove telopeptides, thereby minimizing the risk of immune reactions. Although the likelihood is generally low, materials derived from human or animal sources may carry a theoretical risk of zoonotic disease transmission, which must not be overlooked. Furthermore, regulatory approval—such as that issued by the Korean Ministry of Food and Drug Safety (MFDS)—may differ depending on the number and nature of the product's components, determining whether the formulation is approved for manual injection or requires the use of a delivery device. Accordingly, knowledge of how the main ingredient is sourced and processed is critical for proper clinical application.

**FIGURE 2:**
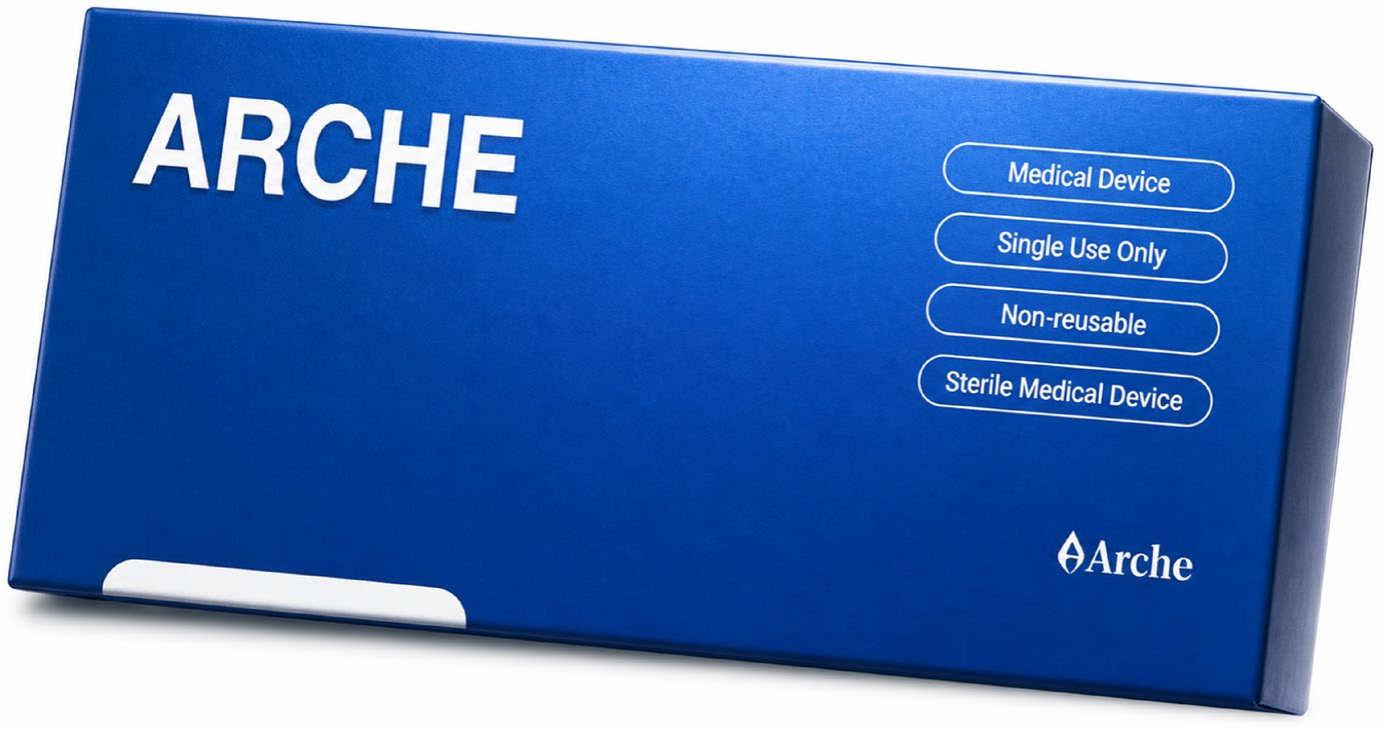
Prior MALDI‐TOF analysis has confirmed that Arche's Ideal Size Chitosan (ISC, Doum Inc., Korea) exhibits a molecular weight predominantly below ~200 Da, indicating a substantial proportion of low‐molecular‐weight chitosan—a form associated with enhanced anti‐inflammatory, antioxidant, and tissue‐regenerative activities. This reduced molecular size enables highly efficient percutaneous absorption across the stratum corneum into the dermis and hypodermis, allowing Arche's ISC technology to effectively deliver bioactive chitosan to deep tissue compartments where collagen synthesis and extracellular matrix remodeling occur, thereby optimizing both therapeutic and aesthetic outcomes.

**FIGURE 3:**
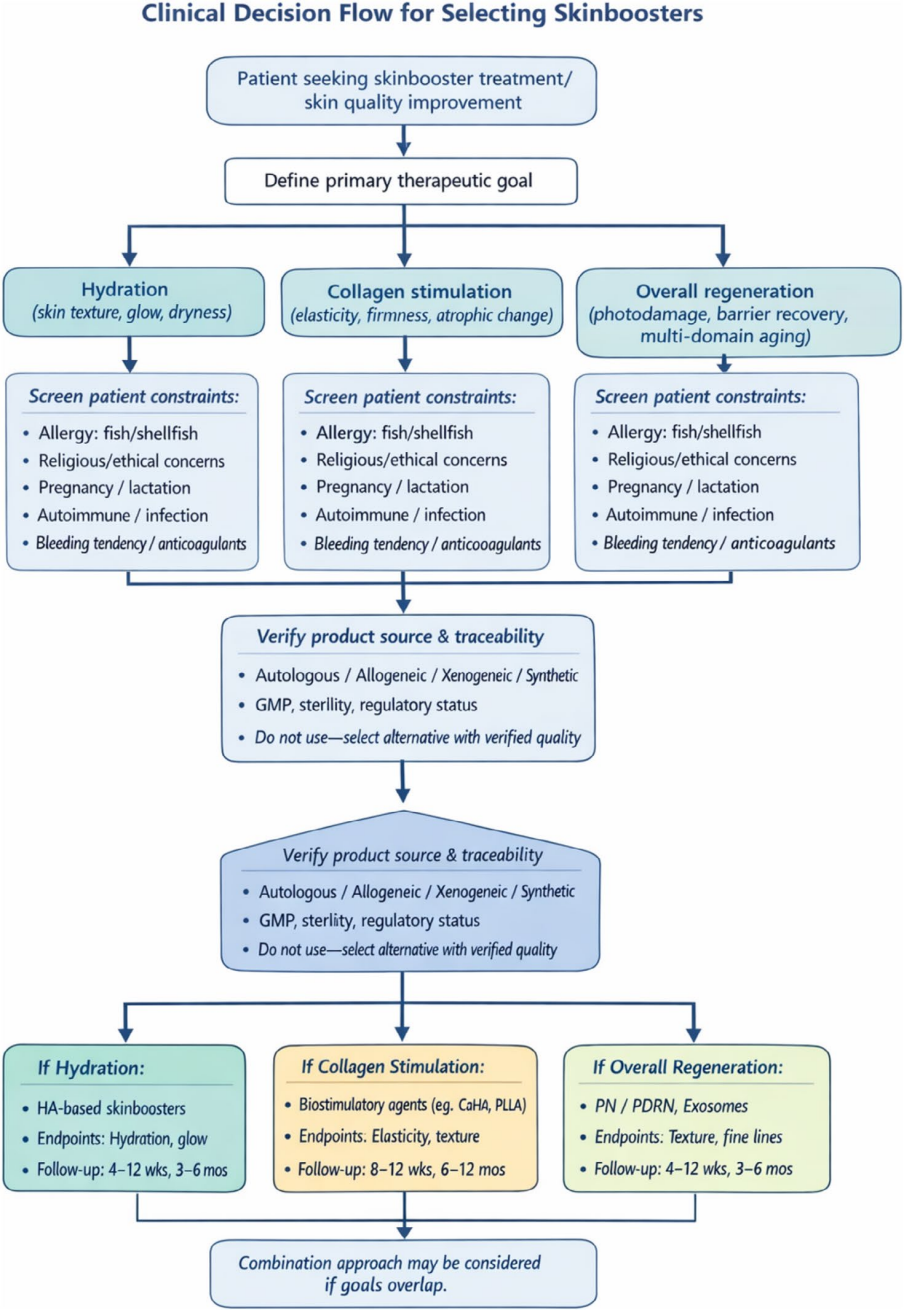
Clinical decision flow for selecting skinboosters in clinical practice. This flow chart operationalizes the proposed classification into a stepwise selection framework. Clinicians first define the primary treatment goal (hydration, collagen stimulation, or overall skin regeneration), then screen patient‐specific constraints (e.g., fish/shellfish allergy, religious/ethical preferences, pregnancy/lactation, autoimmune disease, and active infection/inflammation). Next, product source and traceability are verified (autologous/allogeneic/xenogeneic/synthetic), including manufacturing quality (e.g., GMP and sterility assurance), transparency of labeling (ingredient, concentration, manufacturer), and local regulatory status. Based on these checkpoints, an effect‐aligned skinbooster category is selected, and follow‐up endpoints are defined (e.g., hydration/texture outcomes, elasticity/firmness, or multi‐domain skin quality measures). HA, hyaluronic acid; GMP, Good Manufacturing Practice.

In addition, ethical and religious considerations regarding raw materials vary across countries, and such factors must be taken into account when assessing a product's suitability for international markets. As discussed, skinboosters may be classified into three categories based on their primary effects and five categories based on the origin of their raw materials. Familiarity with this classification system is essential not only for selecting appropriate treatment strategies but also for guiding product positioning in the marketplace and enhancing consumer understanding of the therapeutic approach. To improve clinical usability, we further translate this dual‐axis framework into a stepwise decision algorithm that can be applied at the bedside. Accordingly, Figures 2 and 3 presents a clinical decision flow that begins with defining the primary therapeutic goal, followed by screening patient‐specific constraints (e.g., allergies, pregnancy/lactation, autoimmune or infectious conditions) and verifying product source and traceability (e.g., material origin, GMP/sterility assurance, and local regulatory status). Finally, the algorithm links each goal to an effect‐aligned skinbooster category and practical follow‐up endpoints to guide outcome assessment.

Whereas the origin‐based classification focuses on material source and related safety/ethical considerations, an effect‐based framework helps clinicians match products to the patient's dominant clinical concern. Skinbooster products can also be categorized based on their primary therapeutic effect among the various benefits they offer. Aging skin is largely characterized by a decline in key structural components—most notably hyaluronic acid (HA) and collagen—which leads to diminished skin elasticity and overall deterioration in skin quality. Consequently, many skinbooster formulations are designed to replenish these components of the extracellular matrix (ECM), targeting the restoration of HA and collagen as a central mechanism of action.

In addition to HA and collagen, multiple other factors influence skin quality. Products that contain active ingredients addressing these broader conditions aim to support comprehensive skin regeneration. Thus, the principal effect of such formulations lies in their ability to enhance overall skin health and rejuvenation at a structural and cellular level.

Skinboosters can be categorized into three primary types based on their main therapeutic effects (Figure 1):

1. Hydration‐Based Products: Hydration‐based products represent a significant evolution in dermatological treatments, moving beyond traditional hyaluronic acid (HA) fillers primarily used for volume augmentation to a more diverse application aimed at profoundly improving dermal conditions through enhanced moisture retention and cellular stimulation. At their core, these boosters leverage the remarkable properties of hyaluronic acid, a glycosaminoglycan naturally abundant in the skin's dermal layer, renowned for its unparalleled capacity to bind and retain water molecules—each HA molecule can hold up to 218 water molecules [1], effectively preventing skin dryness and significantly augmenting volume within the dermis and subdermal layers. When injected, HA absorbs moisture from the extracellular matrix (ECM), leading to a substantial expansion of its molecular size (500–1 000 times) [1], which in turn stimulates the elongation of fibroblasts and promotes de novo collagen synthesis. This fibroblast activation is crucial, as it initiates the expression of vital growth factors like connective tissue growth factor, transforming growth factor‐beta1, and transforming growth factor‐beta2, all indispensable for collagen neogenesis, while simultaneously upregulating tissue inhibitor of metalloproteinases genes to inhibit collagen breakdown. Hydration‐based skin boosters come in various forms, including non‐cross‐linked HA, which offers excellent diffusion into peripheral tissues and is ideal for delicate, thin, and dry areas such as around the eyes, and low‐cross‐linked HA, which provides more pronounced volumizing effects and extended duration, making it suitable for other facial regions. Furthermore, advanced formulations, such as Belotero Revive, integrate additional humectants like glycerol, which further amplify skin hydration and fortify the resilience and protective barrier function of mature corneocytes. The comprehensive benefits of these boosters extend beyond mere hydration to include significant antioxidant effects, improved skin elasticity, firmness, and a noticeable glow, with clinical studies demonstrating improvements in skin hydration within weeks, followed by enhanced skin tone and increased epidermal thickness over several months [1, 2]. Despite their efficacy, current limitations include a typical duration of effect of approximately six months following a series of treatments, potential discomfort during injections, and the ongoing challenge of precise delivery to the intended dermal layer, though continuous advancements in injection techniques are expected to mitigate these issues [8, 11]. Representative products include *Restylane Skinboosters Vital Light*, *Skinvive*, *Byryzn*, and *Belotero Revive*.
2. Hydration‐Collagenesis: Hybrid hyaluronic acid (HA) cooperative complexes represent a distinct category of injectable HA‐based skin‐quality treatments, positioned between conventional non‐crosslinked skin boosters and volumizing dermal fillers. These products, such as stable hybrid cooperative complex formulations including Profhilo and regionally marketed products such as Kyloe (Baren Inc., Korea), are generally designed to improve dermal hydration, tissue elasticity, and overall skin quality rather than to provide direct structural augmentation. Their clinical rationale is based on the delivery of stabilized HA complexes that may support prolonged tissue hydration and a favorable extracellular‐matrix environment, leading to perceived improvements in skin firmness, smoothness, and elasticity. Unlike classic fillers, their effect is typically subtle and regeneration‐leaning, making them more suitable for patients seeking global skin‐quality enhancement rather than contour correction. However, product positioning, approved indications, concentration, injection protocol, and marketing claims may differ substantially by country or regulatory region; therefore, clinical use should be guided by local approval status, manufacturer‐supported safety data, appropriate injection depth, and continued post‐market surveillance.
3. Collagen‐Based Products: Collagen‐based skin boosters, while not a single defined product, encompass a variety of treatments and ingredients that primarily function by stimulating the skin's intrinsic collagen production to enhance elasticity, firmness, and overall dermal health [8]. These products aim to replenish collagen, which declines with age. They can be broadly classified into two categories:

- Those that stimulate endogenous collagen production (collagenesis), and
- Those that contain exogenous collagen as a direct ingredient.

From a long‐term perspective, formulations that induce collagen production by triggering mild inflammatory responses and activating fibroblasts within the dermis and subcutaneous tissue are generally considered more effective than those that simply supply collagen transiently. Key agents that promote collagenesis include:

- *Sculptra* (poly‐L‐lactic acid, PLLA)
- *Juvelook* (poly‐D, L‐lactic acid, PDLLA)
- *Lafullen* (polycaprolactone, PCL)
- *UltracoL* (polydioxanone, PDO)
- *Facetem* (calcium hydroxylapatite, CaHA)

Other collagen‐stimulating products developed in Korea include *Aesthefill* (PDLLA), *Olidia* (PLLA), *Collaum* (PLLA), and *Sihler P* (PLLA).

Products containing exogenous collagen include Juveacell (VAIM, Korea), *Colshine*, *Letigen*, *Collaju*, *Essalia*, *Re2O*, and *Celluderm Gen*.

4 Overall Skin Regeneration: The skin is composed of a variety of complex components, including cells, the extracellular matrix (ECM), the dermal–epidermal junction (DEJ)—which links the epidermis and dermis—as well as structural and hydrating elements such as collagen and hyaluronic acid (HA). Skinbooster products that act on these diverse components contribute to comprehensive skin regeneration and repair. The concept of “overall skin regeneration” within the context of skin boosters, as derived from the provided source, refers to a comprehensive improvement in the skin's condition beyond mere hydration or volume augmentation. It encompasses the restoration and enhancement of various dermal and epidermal components, leading to a more youthful, healthy, and resilient skin appearance. This regeneration is achieved through a multitude of mechanisms, primarily by fortifying the ECM, stimulating cellular activity, and addressing signs of aging and damage [8].

Key ingredients commonly found in such formulations include polynucleotides (DOT‐PN, Rejuran), polydeoxyribonucleotides (PDRN), exosomes (Sihler X), collagen, chitosan, secretomes, cytokines, stem cells, various vitamins, and multi‐growth factor complexes (Figure 2).

A precise understanding of the primary therapeutic effect of each skinbooster product is essential not only for clearly communicating the rationale and objectives of the treatment to patients, but also for enabling clinicians to evaluate outcomes based on well‐defined criteria. Therefore, establishing an accurate classification of skin boosters according to their principal effect holds significant clinical importance.

## 3 Skinbooster Agents

PRP and adipose‐derived stem cells (ADSCs) are two prominent autologous materials that have gained significant attention in aesthetic dermatology, particularly for their roles in skin rejuvenation and enhancement. PRP is obtained from the patient's own blood through a process of centrifugation, which concentrates platelets that are rich in growth factors, cytokines, and proteins essential for tissue regeneration and healing. The application of PRP in skin treatments has been shown to improve skin texture, elasticity, and overall appearance by stimulating collagen synthesis and enhancing the extracellular matrix (ECM) [9]. Clinical studies have demonstrated that PRP can effectively reduce wrinkles, improve skin tone, and promote healing in various dermatological conditions, making it a valuable tool in non‐surgical aesthetic procedures. For instance, PRP injections have been associated with significant improvements in skin quality, including increased collagen density and enhanced hydration levels, which contribute to a more youthful appearance [8].

In contrast, ADSCs, which are harvested from adipose tissue, offer a different mechanism of action. These stem cells possess regenerative properties that promote tissue repair and rejuvenation through the secretion of growth factors and cytokines. ADSCs have been shown to enhance skin hydration, increase dermal thickness, and improve overall skin quality by stimulating the production of collagen and elastin [12]. The stromal vascular fraction (SVF) obtained from adipose tissue contains not only stem cells but also a variety of other active cells, including pericytes and immune cells, which contribute to skin regeneration. This multifaceted approach allows for a more comprehensive treatment of skin aging and damage, addressing issues such as volume loss, skin laxity, and pigmentation irregularities [9].

Both PRP and ADSCs have been recognized for their safety and efficacy in clinical applications. PRP, being an autologous product, minimizes the risk of allergic reactions and complications associated with foreign materials. The use of PRP has been associated with minimal side effects, primarily localized to the injection site, such as mild swelling, redness, and discomfort [8]. Similarly, the application of ADSCs is linked to a low incidence of adverse effects, as they are derived from the patient's own body, further enhancing their safety profile [12]. However, the effectiveness of these treatments can vary based on individual patient factors, including age, skin condition, and the specific techniques used for harvesting and application. For example, older patients may experience different outcomes compared to younger individuals due to variations in skin quality and regenerative capacity.

PRP and ADSCs represent significant advancements in the realm of skin boosters, offering promising results for skin rejuvenation. Their autologous nature enhances their safety profile while providing effective solutions for various skin concerns. As research continues to evolve, further studies are needed to optimize treatment protocols, refine harvesting techniques, and fully understand the long‐term benefits of these regenerative therapies [8, 9, 12]. The integration of these innovative approaches into clinical practice holds the potential to revolutionize aesthetic treatments, providing patients with safer and more effective options for achieving youthful and healthy skin.

Human fibroblasts are crucial for maintaining the structural integrity and youthful appearance of the skin by producing essential components of the ECM, such as collagen and elastin. In skin rejuvenation, products derived from cultured human fibroblasts, often sourced from neonatal foreskin, are used. These include conditioned media rich in growth factors and cytokines, referred to as processed skin proteins (PSPs). These PSPs can reduce the appearance of fine lines and wrinkles, improve skin texture, and enhance skin hydration. For instance, products containing PSPs have been shown to improve skin roughness and promote dermal fibroplasia, leading to increased numbers of fibrocytes and fibroblasts. Similarly, conditioned media from neonatal foreskin fibroblast cultures, such as those found in TNS products, contain growth factors and cytokines that promote angiogenesis, modulate inflammation, and enhance ECM deposition, leading to improvements in photodamaged skin [13]. The term “fibroblast growth factor” is also recognized for its role in tissue repair and regeneration [14].

Allogeneic stem cells, particularly mesenchymal stem cells (MSCs), and their derivatives like exosomes, are gaining prominence in regenerative dermatology due to their immunomodulatory and regenerative properties. MSCs can be isolated from various human tissues, including umbilical cord blood or tissue, placenta, and adipose tissue. These cells secrete exosomes, which are nano‐sized extracellular vesicles containing proteins, lipids, and nucleic acids that act as messengers to influence cellular processes in recipient cells [15].

MSC‐derived exosomes (MSC‐EXOs) have demonstrated significant potential in skin rejuvenation and wound healing. They can promote cell proliferation and migration, enhance angiogenesis, and modulate inflammatory responses. For example, MSC‐EXOs have been shown to accelerate the healing of diabetic wounds by promoting M2 macrophage polarization, inhibiting the inflammatory phase, and enhancing collagen deposition. In the context of photoaging, MSC‐EXOs can counteract oxidative stress, reduce the production of matrix metalloproteinases (MMPs), and promote collagen and elastin synthesis by regulating signaling pathways such as NRF2 and MAPK/AP‐1 [15]. Exosomes derived from human pharynx stem cells are also being explored for their exceptional differentiation capabilities, further expanding the potential sources for these therapeutic agents [8].

Secretomes refer to the comprehensive collection of soluble components released by cells into their surrounding environment, including growth factors, cytokines, and exosomes. Dermal fibroblasts, for instance, secrete a diverse array of these molecules that mediate communication with neighboring cells to support ECM maintenance and repair. Many products marketed as “growth factor” products are, in fact, secretome products, which include exosomes. These secretomes can enhance the migration and proliferation of various dermal cells, reduce wrinkle formation, improve skin hydration, and increase collagen synthesis [8].

Cytokines, as signaling proteins, play a vital role in cell communication and immune responses. In skin rejuvenation, cytokines like transforming growth factor‐beta (TGF‐β), interleukin (IL)‐6, and IL‐8 affect collagen biosynthesis and can modulate inflammation [13]. While large molecular size limits their direct penetration, their inclusion in cosmetic formulations, often within secretomes or conditioned media, contributes to their beneficial effects on skin quality.

Collagen, a prevalent natural polymer, is widely used in tissue engineering and skin regeneration due to its unique properties. Historically, injectable collagen products served as dermal fillers. While HA fillers have largely replaced collagen for volume replacement, intradermal collagen injections offer distinct benefits in skin regeneration, including promoting proliferation, biocompatibility, flexibility, and controlled degradation. Innovations like atelocollagen, derived from nonhuman sources with reduced immunogenicity, are specifically designed for intradermal application to enhance aging skin quality. The therapeutic effects of collagen may stem from its inherent properties and its fragmentation into “matrikines,” which can influence ECM remodeling [16].

The integration of components derived from human fibroblasts, allogeneic stem cells, exosomes, secretomes, cytokines, and collagen into skin rejuvenation strategies represents a significant advancement in aesthetic medicine. These allogeneic materials offer diverse mechanisms to improve skin quality, ranging from direct stimulation of ECM production to modulation of cellular processes and inflammatory responses. Continued research, particularly large‐scale controlled studies with objective assessments, is crucial to further elucidate their optimal protocols, combinations, and long‐term efficacy in dermatological practice [8, 13, 14, 15, 16].

Hyaluronic acid remains the most thoroughly researched and widely utilized agent in skin boosting applications. As a naturally occurring glycosaminoglycan abundantly present in the dermal extracellular matrix, HA exhibits remarkable hydrophilic properties, capable of binding water up to 1 000 times its volume, thereby maintaining skin viscoelasticity, hydration, and fiber integrity [16]. The mechanism of action involves stimulation of collagen I synthesis in fibroblasts and enhancement of structural support through mechanical stretching from HA injections, which activates the TGF‐β signaling pathway leading to increased type I collagen production.

Cross‐linked HA‐based skin boosters have been established as the preferred first‐line hydration treatment, demonstrating efficacy both as a standalone therapy and in combination with other agents. Clinical studies have shown that intradermal cross‐linked HA injections significantly improve skin texture, reduce roughness, decrease electrical resistance, and increase dermal thickness by approximately 4% [1, 16]. The interaction of HA with hyaluronan receptors CD44 and CD168 promotes fibroblast migration and proliferation while inhibiting collagenase activity, thereby reducing collagen breakdown and enhancing skin smoothness.

PDRN and PN represent a significant advancement in skin rejuvenation technology. PDRN consists of DNA fragments with molecular weights ranging from 50 to 1 500 kDa, primarily extracted from salmon trout or chum salmon sperm cells, achieving over 95% purity [10]. The mechanism of action involves activation of adenosine A2A receptors, which leads to anti‐inflammatory, anti‐apoptotic, and tissue regenerative effects.

Clinical studies have demonstrated that PDRN promotes angiogenesis, cellular activity, collagen synthesis, and soft tissue regeneration while providing anti‐aging effects [10]. The activation of A2A receptors inhibits NF‐κB and MAPK signaling pathways, blocking the cascade of events initiated by reactive oxygen species that contribute to skin aging. Additionally, PDRN has shown remarkable anti‐melanogenic properties, significantly inhibiting melanin synthesis in a dose‐dependent manner through direct inhibition of tyrosinase activity and reduction of melanocyte‐inducing transcription factor (MITF) expression.

Polynucleotides, consisting of longer nucleotide chains with higher molecular weight compared to PDRN, offer superior viscoelasticity and water‐binding properties. Clinical surveys reveal that 88% of Korean dermatologists use PN injections in their cosmetic practices [17], with studies showing marked improvements in pore size, skin thickness, skin tone, melanin levels, wrinkles, and sagging following intradermal PN injections [17].

Exosomes, the smallest type of extracellular vesicles, ranging from 30 to 110 nm in size, represent a cutting‐edge approach in skin rejuvenation [18]. These vesicles encapsulate proteins, mRNA, miRNA, and lipids within a lipid bilayer derived from cell membranes, contributing significantly to wound healing and skin rejuvenation processes [8]. Despite their abundance in nature, exosomes present significant challenges in extraction and stabilization due to their diminutive size and sensitivity to temperature, pressure, and pH fluctuations [18, 19]. However, clinical evidence remains heterogeneous, and standardization of isolation, characterization, dosing, and long‐term safety is still evolving, with important regulatory implications [18, 19].

The application of exosomes in skin rejuvenation demonstrates remarkable potential in facilitating cellular communication within the epidermis and dermis. These vesicles influence keratinocyte behavior by promoting cellular cohesion and stratification essential for robust skin barrier function. In the dermis, exosomes serve as key messengers influencing fibroblast behavior, enhancing collagen and elastin synthesis, and promoting regenerative capacity for skin anti‐aging [8]. Recent focus has been placed on exosomes derived from the human pharynx, collected through swab‐based sampling, which are recognized for their exceptional differentiation capabilities.

Xenogeneic chitosan, specifically Ideal Size Chitosan (ISC, Doum Inc., Korea), represents a distinctive and highly bioavailable skin boosting agent derived from crustacean or fungal sources. MALDI‐TOF analysis confirms that ISC exhibits a molecular weight predominantly below ~200 Da, indicating a high content of low‐molecular‐weight chitosan, which is associated with enhanced anti‐inflammatory, antioxidant, and tissue‐regenerating properties. Unlike higher‐molecular‐weight chitosan, the reduced size of ISC facilitates superior percutaneous absorption past the stratum corneum to the dermis and hypodermis, allowing it to effectively deliver bioactive chitosan to deep tissue compartments where collagen synthesis and extracellular matrix remodeling occur. Mechanistically, xenogeneic chitosan ISC promotes stem cell homing via upregulation of stromal‐derived factor‐1α, stimulates angiogenesis, and supports fibroblast activity, thereby maximizing both therapeutic and aesthetic efficacy. When compared to cross‐linked HA or polynucleotides, ISC offers the unique advantage of dual functionality: direct tissue regeneration coupled with enhanced transdermal penetration, positioning it as a promising xenogeneic platform for next‐generation skin rejuvenation. Xenogeneic collagen matrices have gained attention as effective skin rejuvenation tools, particularly in post‐surgical reconstruction and tissue regeneration applications. Clinical case studies have demonstrated the successful use of xenogeneic collagen matrices combined with injectable platelet‐rich fibrin for tissue regeneration following surgical procedures [20]. The bilayer structure of these matrices allows for open healing and soft tissue structure filling, promoting enhanced regeneration while providing mechanical coverage and protection.

The biofunctionalization of xenogeneic collagen membranes with autologous platelet concentrates has shown promising results in enhancing angiogenesis and tissue repair. Studies utilizing the yolk sac membrane model have demonstrated that collagen membranes combined with platelet‐rich fibrin induce statistically significant increases in vessel formation and branching points compared to native controls [21]. The pH‐modulating properties of these combinations create optimal conditions for wound healing, with PRF maintaining alkaline pH levels that support tissue regeneration processes.

Xenogeneic MSCs represent a promising frontier in skin rejuvenation, particularly when incorporated into bioprinted hydrogel matrices. Research has demonstrated that biocuratives containing human MSCs associated with hydrogel matrices significantly improve wound healing processes in diabetic [22]. The therapeutic mechanisms involve stimulation of mast cells and M2 macrophages, increased TGF‐β gene expression, and enhanced collagen deposition.

The application of xenogeneic MSCs has shown remarkable effects on the inflammatory profile and regenerative processes. Treatment with MSC‐containing biocuratives accelerated wound healing, improved skin collagen deposition, and increased the number of mast cells in treated areas. The upregulation of IL‐33 gene expression and augmentation of mast cell numbers suggest that the mast cell‐IL‐33 axis plays a crucial role in MSC‐induced wound healing and skin rejuvenation [22].

The integration of these diverse skin boosting agents offers unprecedented opportunities for personalized aesthetic treatments. The combination of different modalities, such as HA with polynucleotides or the incorporation of xenogeneic materials with autologous factors, provides synergistic effects that enhance overall treatment outcomes. Current clinical protocols typically involve multiple treatment sessions spaced 3–4 weeks apart, with effects lasting 6–12 months depending on the specific agent and combination used.

Collagen synthesis stimulants function through a well‐established biological pathway that begins with an initial foreign body reaction upon injection into host tissue. This inflammatory response triggers the body's natural healing mechanisms, initially producing type III collagen during the acute phase. Subsequently, microparticles of the filler material become encapsulated as part of the body's protective response, forming a stable capsule composed primarily of type I collagen that persists as long as the biomaterial remains within the tissue [23].

Two primary synthetic materials dominate the collagen‐stimulating agent category in facial rejuvenation. Calcium hydroxylapatite (CaHA), commercially available as Radiesse, received FDA approval in 2006 and consists of calcium and phosphate ions that occur naturally in human tissue [24], making it bioresorbable. Polycaprolactone (PCL), the active component in Ellansé fillers, received CE approval in 2009 [25], Research has shown that at four months post‐treatment, CaHA produces significantly greater type III collagen compared to HA fillers. Though PCL itself has been an FDA‐approved biomaterial since 2001. This synthetic polymer comprises 70% of Ellansé formulation and is utilized in various medical applications, including biodegradable sutures and orthopedic implants [23].

Current evidence demonstrates that both CaHA and PCL exhibit superior longevity compared to hyaluronic acid (HA) fillers, with studies showing these materials stimulate greater type I collagen production in the long term. Clinical trials have established that CaHA formulations containing lidocaine provide enhanced pain control compared to CaHA alone, while maintaining equivalent efficacy. The safety profiles of both materials are excellent, with minimal side effects reported across multiple studies [23].

Research has shown that at four months post‐treatment, CaHA produces significantly greater type III collagen compared to HA fillers [26]. By nine months, type I collagen staining was higher with CaHA vs. HA, while type III levels decreased, indicating maturation of the collagen synthesis process [26]. Additionally, staining for elastin, cellular proliferation markers, and angiogenesis demonstrated superior results with CaHA at both timepoints [23].

Despite their efficacy, collagen‐stimulating agents present certain limitations that practitioners must consider. Unlike HA fillers, both CaHA and PCL are irreversible treatments that cannot be dissolved with hyaluronidase, which may be viewed as a potential barrier to patient safety. Neither material is appropriate for lip augmentation procedures. Limited evidence exists regarding emergency reversal techniques, with only one documented case of successful Ellansé dissolution using methotrexate [23, 27].

The literature reveals significant gaps in comparative studies between different collagen‐stimulating agents and alternative treatments. Evidence comparing CaHA to PCL, poly‐L‐lactic acid (PLLA), Profhilo, Lotoshon, polymethyl‐methacrylate (PMMA), lipofilling, platelet‐rich plasma, and stem cell therapies remains limited. Current treatment protocols are largely consensus‐based for CaHA and manufacturer‐led for Ellansé, highlighting the need for larger, multicenter randomized controlled trials with histological confirmation of results [23].

While synthetic collagen stimulants demonstrate superior longevity compared to HA fillers, cost‐effectiveness analyses suggest that autologous fat transfer may provide more economical long‐term results. However, this finding requires validation through larger‐scale studies. The clinical applications of collagen‐stimulating agents are best viewed as complementary to rather than competitive with HA fillers, allowing practitioners to select the most appropriate treatment based on individual patient needs and treatment goals [23].

Collagen‐stimulating agents represent a valuable addition to the aesthetic practitioner's armamentarium for facial rejuvenation. Both CaHA and PCL demonstrate safety, efficacy, and superior longevity compared to traditional HA fillers while stimulating natural collagen production. However, their irreversible nature and limited comparative evidence underscore the importance of careful patient selection and the need for continued research through large‐scale, multicenter trials with histological validation to fully establish their role in contemporary aesthetic medicine.

## 4 Conclusion

The field of skin rejuvenation has been revolutionized by the introduction of diverse skin boosting agents, each offering unique mechanisms of action and therapeutic benefits. From the well‐established efficacy of hyaluronic acid to the regenerative potential of polynucleotides and the innovative applications of xenogeneic materials, these treatments provide comprehensive solutions for addressing age‐related skin changes. The systematic classification of skinboosters into five material‐based categories (autologous, allogeneic, xenogeneic, synthetic, and compound formulations) and three effect‐based categories (hydration‐based, collagen‐based, and overall skin regeneration) provides clinical practitioners with a robust framework for treatment selection and patient counseling. This dual classification system enables clinicians to make informed decisions based on both the source material properties—which determine safety profiles, regulatory considerations, and potential contraindications—and the primary therapeutic objectives required for each individual patient's skin concerns.

The integration of these diverse skin boosting agents offers unprecedented opportunities for personalized aesthetic treatments, with current clinical protocols demonstrating that combination approaches often provide synergistic effects that enhance overall treatment outcomes. The evolution of delivery methods, including needle‐free jet injectors and automated multi‐needle systems, has improved patient comfort and treatment precision, while technological advances combined with expanding understanding of cellular mechanisms underlying skin aging and regeneration position skin boosters as a cornerstone of modern aesthetic dermatology. However, significant gaps remain in the current evidence base, particularly in comparative studies between different collagen‐stimulating agents and alternative treatments, with current treatment protocols being largely consensus‐based rather than supported by large‐scale, multicenter randomized controlled trials with histological validation.

Moving forward, the continued advancement in understanding cellular mechanisms, delivery technologies, and combination therapies promises to further enhance the effectiveness and safety of skin rejuvenation treatments, offering patients increasingly sophisticated options for maintaining healthy, youthful‐appearing skin. Future research should focus on optimizing treatment protocols, understanding long‐term effects, and developing novel combinations that maximize therapeutic benefits while maintaining excellent safety profiles. As the field continues to evolve, the establishment of this comprehensive classification system will serve as a foundation for evidence‐based practice, regulatory framework development, and the advancement of skin booster technology in aesthetic medicine.

## Author Contributions

All authors have reviewed and approved the article for submission. Conceptualization, Gi‐Woong Hong, Kyu‐Ho Yi. Writing – Original Draft Preparation, Isabella Rosellini, Kyu‐Ho Yi. Writing – Review and Editing, Hyunggyu Kang, Olena Sydorchuk. Visualization, Gi‐Woong Hong. Supervision, Kyu‐Ho Yi.

## Funding

The authors have nothing to report.

## Disclosure

The authors have nothing to report.

## Ethics Statement

Ethical approval was not required because this article is a review/classification article and did not involve human participants or animal experiments. This article is a review of previously published studies and does not involve any original research involving human participants or animals conducted by the authors. Therefore, ethical approval was not required.

## Consent

No new human data were generated or analyzed in this study. Informed consent for publication is not applicable.

## Conflicts of Interest

The authors declare no conflicts of interest.

## Data Availability

The data that support the findings of this study are available from the corresponding author upon reasonable request.
